# Editorial: Eye-tracking while reading for psycholinguistic and computational models of language comprehension

**DOI:** 10.3389/fpsyg.2023.1326408

**Published:** 2023-11-28

**Authors:** Marijan Palmović, Lena A. Jäger, Nora Hollenstein

**Affiliations:** ^1^Laboratory for Psycholinguistic Research, University of Zagreb, Zagreb, Croatia; ^2^Department of Computational Linguistics, University of Zurich, Zürich, Switzerland; ^3^Center for Language Technology, University of Copenhagen, Copenhagen, Denmark

**Keywords:** eye-tracking, reading, computational modeling, language comprehension, natural reading

## 1 Aim of this Research Topic

Eye-tracking is a powerful technology for studying language processing. In recent years, it has been employed increasingly for reading studies based on collecting and analyzing reading corpora obtained in a natural setting, i.e., based on texts not experimentally manipulated as stimuli in minimal pairs. Typically, these texts are tagged with fixation and saccades data and some linguistic or psycholinguistic parameters. The large amount of the corpus data allows for new analytical techniques, resulting in new insights into psycholinguistic accounts of reading and, more generally, in psycholinguistic and computational models of language comprehension. Finally, collecting and sharing such corpora in various languages facilitates cross-linguistic studies of psycholinguistic phenomena, bilingual or multilingual studies, and research into individual differences among readers.

The creation of reading corpora allows for new directions in psycholinguistics research. It is the aim of this Research Topics to provide a platform for a discussion on this development in several directions:

1. The theoretical implications of large eye-tracking reading data in psycholinguistics;
2. Opportunities for comparative (cross-linguistic) and bilingual (multilingual) studies of psycholinguistic phenomena relevant to the formulation or evaluation of psycholinguistic or computational models of language comprehension;
3. The inclusion of languages other than English in order to alleviate the English language bias in psycholinguistic research.

In addition, methodological considerations within the eye-tracking research (e.g., corpus vs. experimental data) and between eye-tracking and similar methods (e.g., self-paced reading) reflect many issues in contemporary psycholinguistic modeling of language comprehension such as the interpretation of the processes captured by some dependent variable obtained by eye-tracking or discussion about the arguments corroborating or refuting a particular psycholinguistic model. Finally, one expects that the reading corpora would allow for a more comprehensive study of the individual differences among readers, an issue that has recently attracted considerable attention in eye-tracking research.

## 2 Statistics of this Research Topic

The Research Topic was open from 27/06/2022 and the extended deadline concluded on 16/01/2023. Seventeen articles were submitted within this period, of which 11 were accepted after careful peer-reviewing.

## 3 Summary of this Research Topic

This Research Topic includes studies of a wide range of languages, including multiple language families (Sino-Tibetan, Indo-European, and Nortwest-Caucasian), various scripts (Latin and Cyrillic alphabets, Chinese characters), and modalities (written and signed languages). From a methodological perspective, the studies accepted in this Research Topic can be split into works concerning the computational modeling of reading and psycholinguistics investigations of language comprehension.

The computational articles focus on a diverse range of topics from predicting reading tasks (Hollenstein et al.), predicting metrics extracted from eye-tracking data (Salicchi et al.), to the acceptability of machine translation technology (Kasperė et al.), and using machine learning to extract neural components during rapid automatized naming (RAN) tests (Christoforou et al.).

The psycholinguistic articles in this Research Topic study a number of factors relevant to improving our understanding of reading comprehension, including lexical access (Chang et al.), grammatical errors (Søby et al.), individual differences (Gong and Shuai), typological differences (Zdorova et al.), text formatting (Medved et al.), and exposure to sign language (Ziubanova et al.). In the following, we briefly describe the contributions of each article.

Firstly, on the computational side, Christoforou et al. propose a novel machine-learning-based algorithm that extracts neural components from EEG and eye-tracking recordings of children with and without dyslexia during serial rapid automatized naming (RAN) tests. The authors show that these components capture the neural activity of cognitive processes associated with naming speed and are informative of group differences.

The ZuCo corpus contains eye-tracking and EEG data during normal reading and information-searching reading in English. The benchmark provides a new hidden testset for machine learning models trained to distinguish these two tasks (Hollenstein et al.). Improving the performance of reading task classification will be useful in identifying the relevant features and can advance models of reading.

Previous research in computational linguistics has investigated whether distributional language models can predict metrics extracted from eye-tracking data. In their study, Salicchi et al. propose a regression experiment for estimating different eye-tracking metrics on two English corpora, contrasting the quality of the predictions with and without the surprisal and the relatedness components. Their results suggest that both components play a role in the prediction, with semantic relatedness surprisingly contributing also to the prediction of function words.

Between the realms of computational language processing and psycholinguistics, Kasperė et al. leverage eye-tracking to investigate the acceptability of machine translation technology between professional translators and non-professionals. In a study in which participants read an English text machine-translated into Lithuanian, the authors analyze whether raw machine translation output is processed in the same way by both groups. In terms of acceptability overall, professional translators critically assess machine translation on all components, which confirms the findings of previous similar research. However, the current study draws attention to the lower awareness of non-professionals regarding machine translation quality.

On the side of psycholinguistic reading research, Chang et al. employ an eye-tracking experiment to corroborate the “graded pre-activation” account of lexical access in explaining the predictions of the coming words in a sentence.

Grammar errors are a natural part of everyday written communication and come in different forms, e.g., syntactic errors, morphological agreement errors, and orthographic errors. Søby et al. examine whether some types of naturally occurring errors attract more attention than others during the reading of Danish texts, measured by detection rates. While this study did not measure eye movements, the differences in error detection patterns point to shortcomings of existing eye-tracking models.

Furthermore, in a sentence reading eye-tracking study, Gong and Shuai assess participants' reading skills on a number of language and cognitive measures while manipulating the lexical properties of the words in the stimulus sentences. The interactions between text properties and reading skills proved to be significant on early and late eye-tracking measures.

Tywoniw analyzes reading strategies in English as L2 with participants of varying native language backgrounds. Their individual differences and the differences across experimental conditions (close reading, multiple-choice, and reading-to-summarize) are studied to identify the predictors of reading behavior.

Zdorova et al. study how typological differences impact reading behavior of Adyghe-Russian bilinguals. A robust frequency effect was found in Adyghe, while the words of the same length in Adyghe and Russian were read slower in Adyghe due to their complex morphological structure.

Not only linguistic characteristics but also visual aspects influence reading comprehension. More and more educational material is delivered to students through digital screens. Therefore, the text format in which these materials are presented is an important aspect to consider. Medved et al. investigate the effect of letter shape on readers' feelings of pleasantness during reading, reading fluency, and text comprehension and memorization of Slovenian texts. They find that softer typefaces of rounder shapes should be used in educational materials for a more pleasant reading experience and improved learning process.

Finally, Ziubanova et al. study the benefits of early exposure to spoken and sign language for deaf adults and adults with severe hearing impairments in an eye-tracking sentence reading experiment. The benefits of early exposure were confirmed for adults with severe hearing impairments.

## Author contributions

NH: Writing - original draft, Writing—review & editing. MP: Writing—original draft, Writing—review & editing. LJ: Writing—original draft, Writing—review & editing.

